# A population-level study into health vulnerabilities of mothers and fathers involved in public law care proceedings in Wales, UK.

**DOI:** 10.23889/ijpds.v7i3.2003

**Published:** 2022-08-25

**Authors:** Rhodri Johnson, Laura North, Bachar Alrouh, Ann John, Kerina Jones, Ashley Akbari, Jon Smart, Simon Thompson, Claire Hargreaves, Stefanie Doebler, Linda Cusworth, Karen Broadhurst, David Ford, Lucy Griffiths

**Affiliations:** 1 Swansea University; 2 Lancaster University

## Objectives

Under section 31 (s.31) of the UK Children Act 1989, public law care proceedings can be issued if there is concern a child is subject to, or at risk of significant harm. We examined health vulnerabilities of parents involved in public law care proceedings in the two-year period prior to involvement.

## Approach

Our study created an anonymised individual-level population-based cohort, with a matched comparison group of parents in Wales who were not subject to care proceedings, matched on age, sex and deprivation. Family court data provided by Cafcass Cymru were linked to population-level healthcare records held within the Secure Anonymised Information Linkage (SAIL) Databank. Demographic characteristics, overall health service use and health profiles of parents of children subject to s.31 care proceedings between 2011 and 2019 were examined.

## Results

Data were available for 8,821 parents involved in care proceedings between 2011 and 2019, with a comparison group of 32,006 parents. Nearly half (47.6%) of cohort parents resided in the most deprived quintile. Higher levels of healthcare use were found for cohort mothers and fathers compared to the comparison group across multiple healthcare settings, with the most pronounced differences for emergency department attendances (59.3% vs 37.0%). Health conditions with the largest variation between groups were related to mental health (43.6% vs 16.0%), substance use (19.4% vs 1.6%) and injuries (41.5% vs 23.6%).

## Conclusion

This study highlights the heightened socioeconomic and health vulnerabilities of parents who experience care proceedings concerning a child. Better understanding of the needs and vulnerabilities of this population may provide opportunities to improve a range of support and preventative interventions that respond to crises in the community.

